# Design, Synthesis, Anticancer Evaluation and Molecular Modeling of Novel Estrogen Derivatives

**DOI:** 10.3390/molecules24030416

**Published:** 2019-01-24

**Authors:** Abd El-Galil E. Amr, Elsayed A. Elsayed, Mohamed A. Al-Omar, Hanan O. Badr Eldin, Eman S. Nossier, Mohamed M. Abdallah

**Affiliations:** 1 Pharmaceutical Chemistry Department, Drug Exploration & Development Chair (DEDC), College of Pharmacy, King Saud University, Riyadh 11451, Saudi Arabia; malomar1@ksu.edu.sa; 2 Applied Organic Chemistry Department, National Research Center, Cairo, Dokki 12622, Egypt; 3 Zoology Department, Bioproducts Research Chair, Faculty of Science, King Saud University, Riyadh 11451, Saudi Arabia; eaelsayed@ksu.edu.sa; 4 Chemistry of Natural and Microbial Products Department, National Research Centre, Cairo, Dokki 12622, Egypt; 5 Chemistry Department, Faculty of Science, King Khalid University, MahailAsir 61421, Saudi Arabia; hanan.oamr@gmail.com; 6 Department of Dyeing, Textile Industrial Division, National Research Center, Cairo, Dokki 12622, Egypt; 7 Pharmaceutical Medicinal Chemistry Department, Faculty of Pharmacy (Girls), Al-Azhar University, Cairo 11754, Egypt; dr.emannossier@gmail.com; 8 Atos Pharma, Elkatyba Land, Belbis 44621, El-Sharkya, Egypt; mmostafa201120@yahoo.com

**Keywords:** estrogen derivatives, design, anticancer evaluation, molecular modeling studies

## Abstract

A series of estrone derivatives **3**–**8** was designed and synthesized using estrone arylmethylenes **2a**,**b** as starting materials and their structures were confirmed by different spectral data and elemental analyses. All the newly synthesized compounds exhibited potent in vitro and in vivo cytotoxic activities against breast cancer cell lines. In addition, all compounds were subjected to in vitro and in vivo inhibition assays for EGFR and VEGFR-2 kinases as well as p53 ubiquitination activity to obtain more details about their mechanism of action. Based on the promising results, a molecular docking study was investigated for the most representative compound **5a** against the two targets, EGFR and VEGFR-2 kinases, to assess its binding affinity, hoping to rationalize and obtain potent anticancer agents in the future.

## 1. Introduction

Breast cancer is the most common malignant cancer for women, affecting about 1.7 million patients and causing 0.52 million deaths in 2012 [1]. Different factors may contribute to this encumbrance of breast cancer including genetics, life styles and environmental factors. The mechanism of breast cancer involved in cell proliferation, invasion and metastasis is not fully resolved [2,3,4]. In spite of enormous efforts in research to detect and treat cancer, discovery of optimal cancer therapies is still difficult due to the severe side effects associated with chemotherapeutics, as well as appearance of tumor resistance [5]. Recently, discovery of anticancer drugs has been stimulated from conventional cytotoxic drugs to those with multi-targeted mechanisms to produce potent anticancer agents with minimal side effects [6].

Receptor tyrosine kinases (RTKs), epidermal growth factor receptor (EGFR) and vascular endothelial growth factor receptor-2 (VEGFR-2) play vital roles in regulating tumor cell proliferation, differentiation, survival, angiogenesis and apoptosis [7,8,9]. Moreover, the tumor suppressor p53 is important for protection of mammalian cells against genetic damage and any dysfunction of it leads to evidence of cancer [10]. There is a link between p53 and the oncogene HDM2 in a negative feed-back loop in which p53 activates HDM2. The latter acts as a p53-specific ubiquitin E3 ligase and thus promotes degradation of the p53 protein through the ubiquitin–proteasome system [11]. Aberrations in p53 regulation are frequently observed in tumors through overexpression of the p53 negative regulator HDM2 [12]. These RTKs and p53 ubiquitination have been successfully explored as attractive targets for antitumor therapies, particularly for developing multi-target anticancer drugs with improved therapeutic efficacies.

Estrogens are important hormones in the female body and are biosynthesized in human body from cholesterol [13]. These steroids interact with multiple organ systems and have a major impact on the physiological events that occur through a woman’s life. Estrogens play a crucial role in cell proliferation; however, their overexpression stimulates excess proliferation of hormone sensitive cells, leading to various types of hormone-dependent cancers such as breast, uterine, ovarian, prostate and endometrial cancers [14].

**Figure 1:**
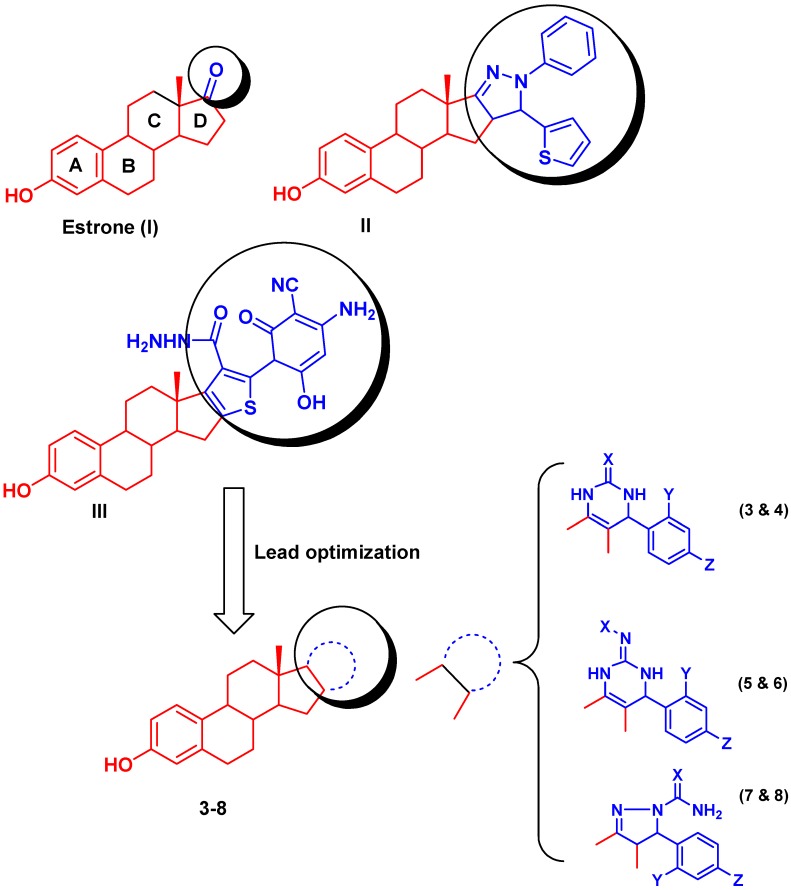
Lead optimization of estrone **I** and other known derivatives (**II** & **III**) showing promising cytotoxic activity against the MCF-7 cancer cell line.

Fusion of steroid molecules with heterocycles at the D ring of the steroid nucleus has been of pharmaceutical interest [15,16,17,18]. Thus, lead optimization of estrone-based anticancer agents (**I**–**III**) needs a lack of estrogenic activity through either opening of ring D or inversion at C-17 of the estrane skeleton [19,20,21,22,23,24,25] (Figure 1).

In view of these reports and in continuation of our interest in the chemical and pharmacological properties of substituted heterocyclic derivatives [26,27,28,29,30,31,32], we report herein the synthesis of a new series of estrones fused with substituted pyrimidine and pyrazoline rings. The biological activities of the newly synthesized products were studied in vitro and in vivo against breast cancer, EGFR and VEGFR-2 kinases, and p53 ubiquitination. A molecular docking study has also been conducted to validate the findings.

## 2. Results and Discussion

### 2.1. Chemistry

**Scheme 1:**
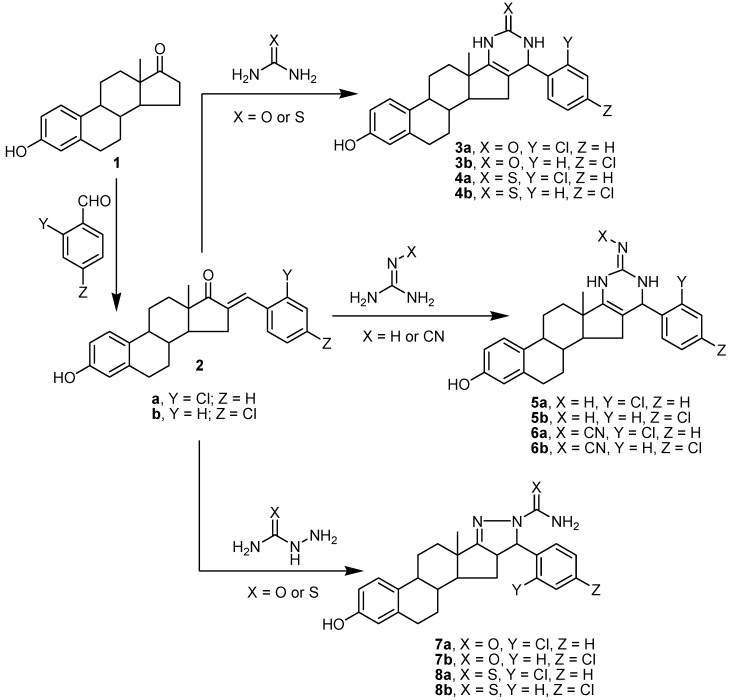
Synthetic pathway for compounds **3**–**8**.

Arylmethylene of estrone derivatives **2a**,**b** [33,34,35] was synthesized via Aldol condensation of estrone **1** and 2-chlorobenzaldehyde or 4-chlorobenzaldehyde in ethanol in the presence of 30% potassium hydroxide according to the previous reported procedure. Treatment of compounds **2a**,**b** with urea or thiourea, in the presence of sodium ethoxide afforded the corresponding 2-oxopyrimidines **3a**,**b** and 2-thioxopyrimidines **4a**,**b**, respectively. Additionally, condensation of **2a**,**b** with guanidine hydrochloride or 2-cyanoguanidine, in the presence of sodium ethoxide afforded the corresponding 2′-aminopyrimidines **5a**,**b** and 2′-cyanoiminopyrimidines **6a**,**b**, respectively. Finally, reaction of **2a**,**b** with semicarbazide or thiosemicarbazide in refluxing dioxane afforded the corresponding 1-carbamoylpyrazolines **7a**,**b** and 1-thiocarbamoylpyrazolines **8a**,**b**, respectively (Scheme 1).

### 2.2. Biological Evaluation

#### 2.2.1. In Vitro Cytotoxic Activity against MCF-7 Cancer Cells

**Figure 2:**
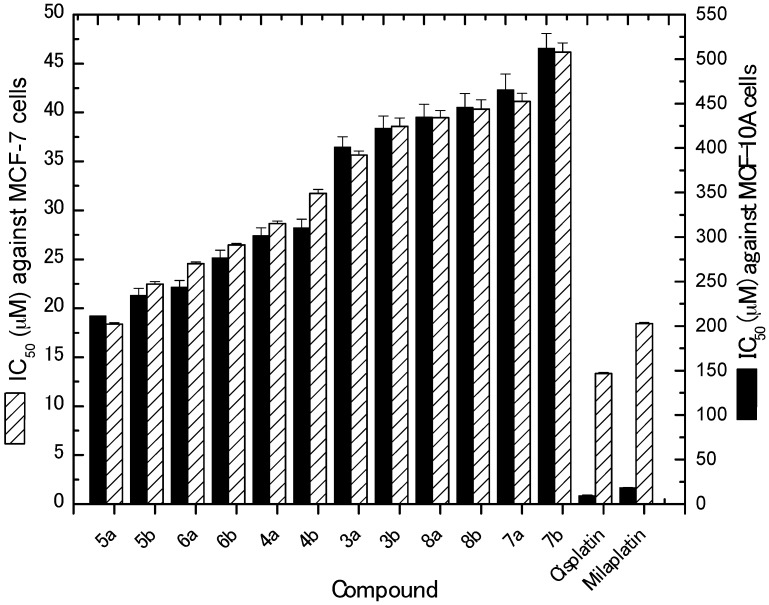
In vitro cytotoxicity activities of the tested compounds against the MCF-7 cancer cell line.

In vitro cytotoxic activities of the newly prepared derivatives toward MCF-7 cells were assessed. All the tested compounds showed potential cytotoxic activities towards MCF-7, at a micro molar level (Table 1, Figure 2). However, the descending order of activity was as follow **5a**, **5b**, **6a**, **6b**, **4a**, **4b**, **3a**, **3b**, **8a**, **8b**, **7a** and **7b**. Moreover, the most active compound (**5a**) was 2.5-fold more toxic than the least active one (**7b**) towards the MCF-7 cell line (IC_50_ = 18.38 ± 0.13 and 46.17 ± 0.93 µM, respectively). The descending activities of the different prepared derivatives may be correlated with their structures. Generally, it can be observed that pyrimidine-containing derivatives (**3**–**6**) are more active than those containing pyrazoline (**7**, **8**). Furthermore, substitution at p-2 on the pyrimidine moiety can affect the cytotoxic activity in descending order by different groups as follows: imino (**5a**, **5b**) > cyanoimino (**6a**, **6b**) > thioxo (**4a**, **4b**) > oxopyrimidine (**3a**, **3b**). On the other hand, the compounds bearing thiocarbamoyl pyrazoline fragments (**8a**, **8b**) exhibited better activity than those bearing carbamoyl pyrazoline (**7a**, **7b**). Finally, by screening the results of all tested compounds, it was noticed that derivatives having a 2-chlorophenyl moiety at p-6 of pyrimidine revealed higher potency than those having 4-chlorophenyl. Comparing IC_50_ values obtained for the synthesized derivatives against MCF-7 with those obtained against non-tumorigenic MCF-10A cells, we can concluded that the synthesized derivatives have much less toxicity against normal cells. Generally, it can be seen that the IC_50_ values obtained for normal non-tumorigenic MCF-10A cells were about 9.78 to 11.49-fold higher than those obtained by MCF-7 cells.

#### 2.2.2. In Vivo Anti-Breast Cancer

**Figure 3:**
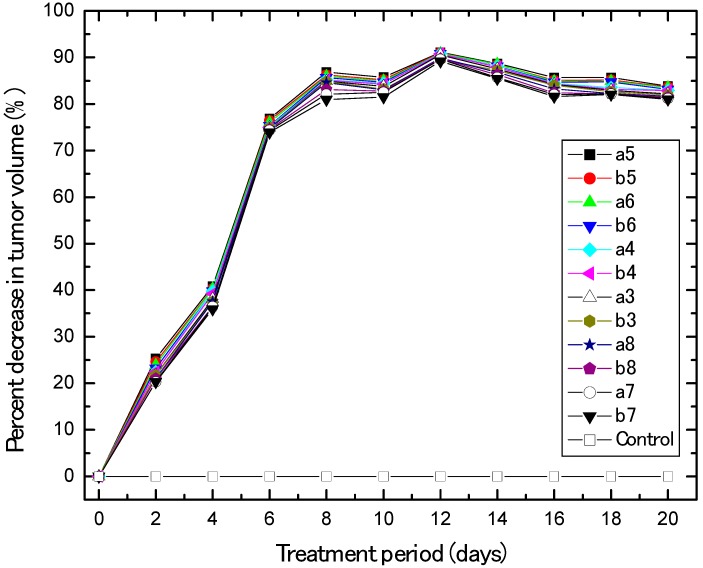
Percentage of decrease in tumor volume as affected by different synthesized compounds.

The in vivo anti-breast cancer activities of different synthesized derivatives were evaluated using a breast cancer mouse xenograft model. Figure 3 shows data obtained for inhibition percentages of tumor growth, i.e., decrease in tumor growth, after exposure to prepared derivatives for the whole period of experiment, in comparison to tumor development in control animals. Results revealed that all synthesized compounds showed potential inhibitory effects on tumor growth upon treatment from day 2. Furthermore, the degree of inhibition in tumor growth increased gradually over time until reaching maximal inhibition after 12 days; afterwards, inhibition percentages slightly decreased at 14 days, and then remained more or less constant for the rest of treatment period. Compound **5a** showed the most promising effect in terms of growth inhibition, where tumor growth decreased by about 25.36% after only 2 days of treatment, and maximal inhibition of 91.1% was recorded after 10 days of treatment. After 14 days, the inhibition percentage decreased to 88.7% and then remained more or less constant up to 20 days of treatment. It can be observed that there is agreement between in vivo inhibitory patterns of the different derivatives and their in vitro anticancer patterns of activity. Also, the newly synthesized estrone derivatives described here coincide with those reported earlier [24]. Furthermore, estrone derivatives have been reported to inhibit in vivo tumor growth in a dose-dependent manner [36,37] through their inhibitory action on 17-hydroxysteroid dehydrogenase.

#### 2.2.3. In Vivo and In Vitro Inhibition of p53 Ubiquitination Activities

p53 was found to play an important role in cancer prevention as a suppressor protein through variable pathways. Binding of p53 to E3 ubiquitin protein ligase HDM2 results in inhibiting its ability as a transcription activator, i.e., a negative regulatory mode of action. It was postulated that blocking p53 binding site on HDM2 is useful in obtaining potential antitumor agents. However, there are few reports on scaffolds having inhibitory HDM2 activity. Murine Double Minute 2 (MDM2) is a widely studied regulator that is used to inhibit p53 activity either by direct binding or by acting as an ubiquitin ligase (E3) catalyzing p53 ubiquitination and proteasome-mediated degradation [38].

**Figure 4:**
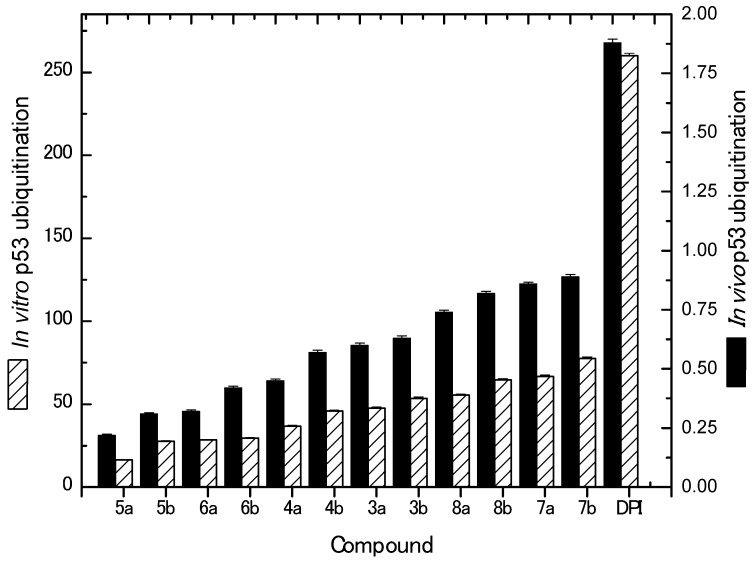
IC_50_ (µM) values of in vitro and in vivo p53 ubiquitination of the newly synthesized compounds.

All newly synthesized compounds exhibited in vitro suppression of p53 ubiquitination when incubated with GST-tagged HDM2, p53, ubiquitin or E1 and E2 (UbcH5B) ligases (IC_50_ ranged from16.45 ± 0.23 to 77.56 ± 0.97 µM). Additionally, the evaluated compounds revealed excellent in vivo inhibition of p53 ubiquitination, with IC_50_ ranging from 0.22 ± 0.0043 to 0.89 ± 0.0099 µM. By comparing the results with the standard diphenyl imidazole drug (Table 2, Figure 4), it was noticed that all tested compounds represented excellent and more potent activity than the reference for in vitro and in vivo inhibition of p53 ubiquitination with a descending order of activity as follow **5a**, **5b**, **6a**, **6b**, **4a**, **4b**, **3a**, **3b**, **8a**, **8b**, **7a** and **7b**. Also, compound **5a** displayed the highest activities, which were 15.8- and 8.6-fold more active than the standard drug for in vitro and in vivo inhibition of p53 ubiquitination, respectively.

#### 2.2.4. Inhibition of EGFR and VEGFR-2 Kinases

**Figure 5:**
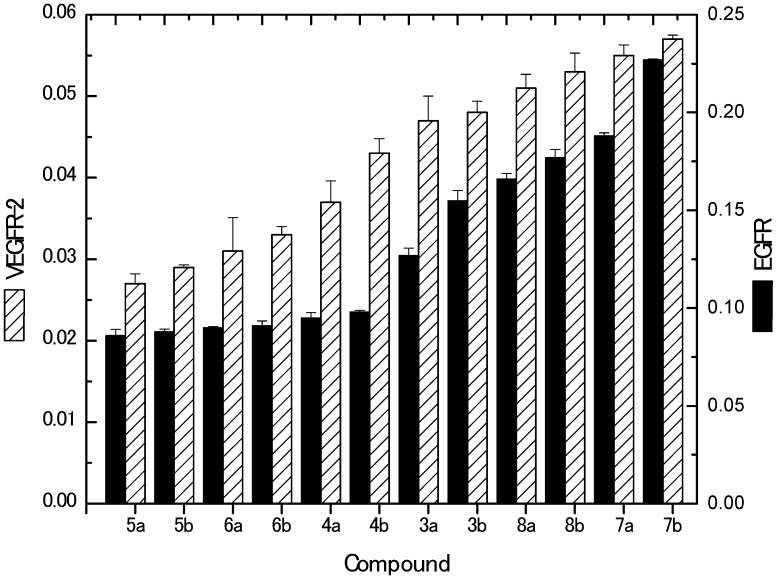
IC_50_ values (µM) of EGFR and VEGFR-2 enzyme inhibition caused by the synthesized compounds.

The same list of the tested compounds was screened for their in vitro inhibition activity against EGFR and VEGFR-2 kinases. IC_50_ values are reported in Table 3, Figure 5 and were compared with the positive control drug delphinidin. All examined compounds efficiently inhibited EGFR and VEGFR-2 kinases in a dose-dependent manner, with IC_50_ ranging from 0.086 ± 0.0032 to 0.227 ± 0.0004 µM for EGFR and from 0.027 ± 0.0012 to 0.057 ± 0.0005 µM for VEGFR-2 while compound **5a** turned out to be most potent micromolar inhibitor. It was observed that the inhibitory activities for the new derivatives had a similar behavior in terms of descending order as **5a**, **5b**, **6a**, **6b**, **4a**, **4b**, **3a**, **3b**, **8a**, **8b**, **7a** and **7b** (Figure 5) and had higher activities than the reference drug against both enzymes (IC_50_ of delphinidin = 6.27 ± 0.00076 µM against EGFR and 5.09 ± 0.0012 µM against VEGFR-2). Furthermore, the most potent derivative **5a** was about 188.5- and 72.9-fold more active than delphinidin against EGFR and VEGFR-2 kinases, respectively.

### 2.3. Molecular Modeling Studies

To explain and rationalize the experimental results obtained, molecular docking studies were conducted on the representative compound **5a** using Molecular Operating Environment (MOE, 10.2008) software [39] against two targets, EGFR (PDB code: 1M17) [40] and VEGFR-2 (PDB code: 4ASD) [39] binding site structures. The root mean square differences (RMSD) between the top docking poses and original crystallographic geometry of co-crystallized ligands erlotinib for EGFR and sorafenib for VEGFR-2 were 0.90 and 0.88 A°, respectively.

**Figure 6:**
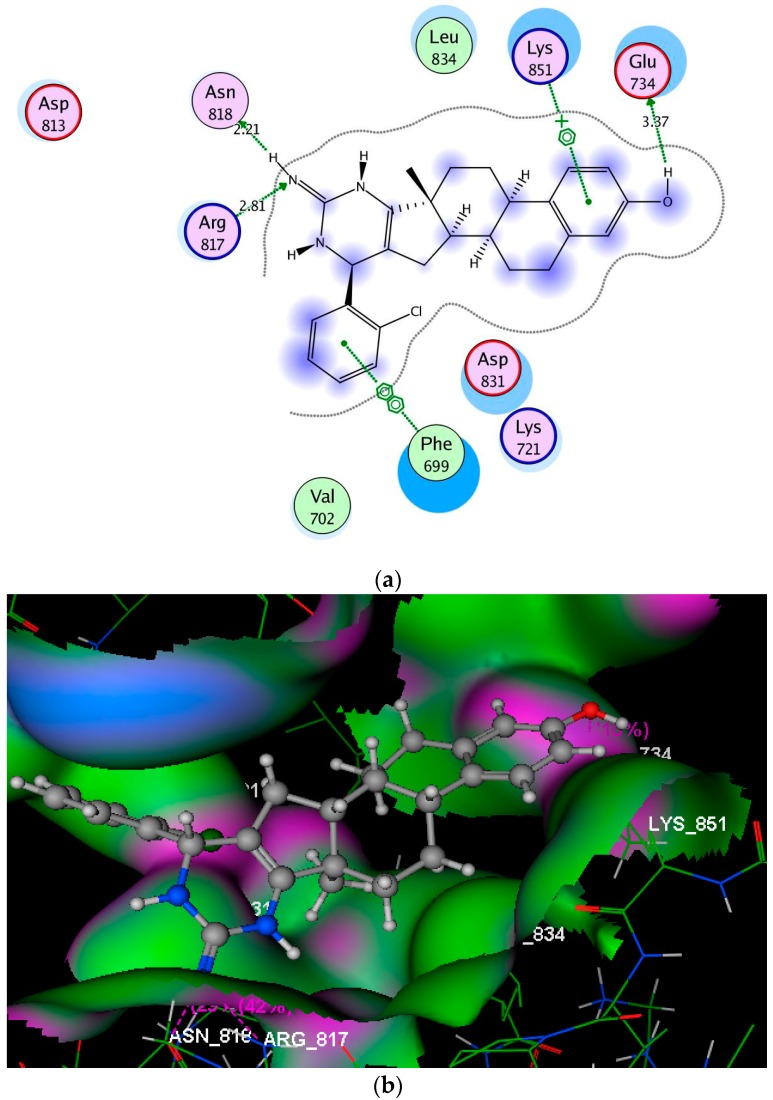
(**a**,**b**) images show 2D and 3D graphs of compound **5a** docked into EGFR binding site of protein (1M17). Green color indicates the hydrophobic area, pink color indicates a high polar area, blue color indicates a mild polar area and dotted lines and arrows represent hydrogen bonds.

From Figure 6 and regarding the EGFR target, compound **5a** showed two hydrogen bonds between the imino group at p-2 of the pyrimidine moiety and the sidechains of **Arg817** and **Asn818** (distance: 2.81 and 2.21 Å, respectively). Furthermore, the acidic **Glu734** of the receptor formed a consistently stable hydrogen bond with the terminal hydroxyl group (distance: 3.37 Å). Moreover, compound **5a** displayed two critical hydrophobic interactions, one between the centroid of 4-chlorophenyl and **Phe699** as an arene-arene interaction and the other between the centroid of ring A of the steroidal moiety and **Lys851** as an arene-cation interaction.

**Figure 7:**
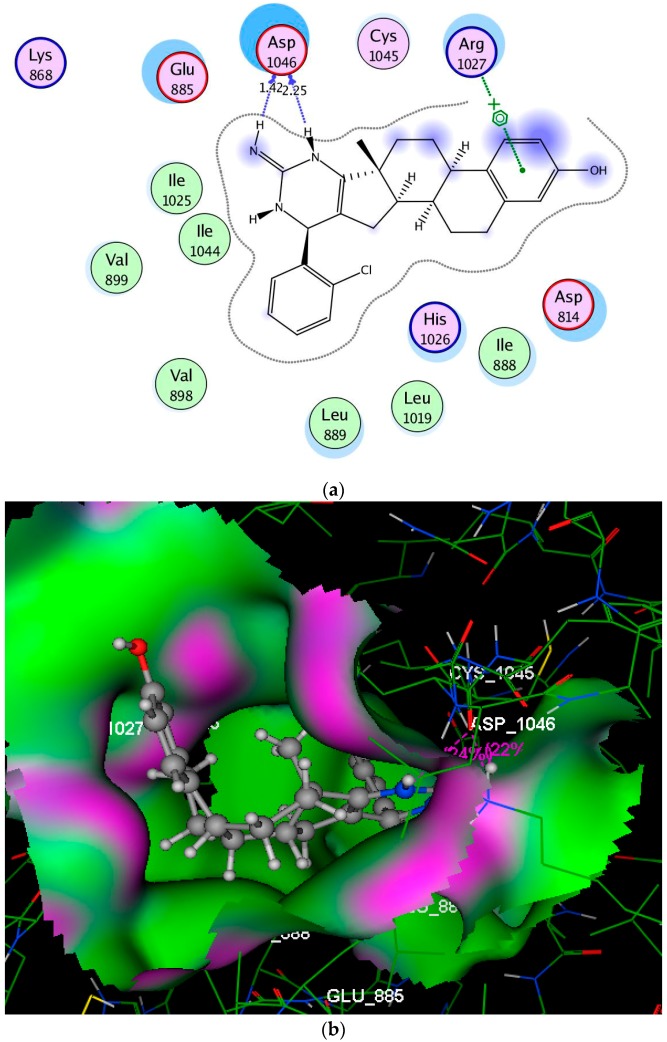
(**a**,**b**) images show 2D and 3D graphs of compound **5a** docked into VEGFR-2 binding site of protein (1M17). Green color indicates a hydrophobic area, pink color indicates a high polar area, blue color indicates a mild polar area and dotted lines and arrows represent hydrogen bonds.

A docked model of **5a** inside VEGFR-2 is shown in Figure 7. The pyrimidine ring was engaged in two hydrogen bond donors between N1 and the imino group at p-2 with the backbone of **Asp1046** (distance: 2.25 and 1.42 Å, respectively). Additionally, the centroid of ring A of the steroidal scaffold was involved in an arene-cation interaction with the basic **Arg1027**.

The molecular docking results indicated that compound **5a** was accommodated well in the binding sites of both enzymes through ring A of the steroidal scaffold and the pyrimidine moiety linked to ring D. Thus, the current results demonstrated that the estrone-pyrimidine hybride structure is a considerable scaffold for subsequent optimization to develop novel effective inhibitors EGFR and VEGFR-2 kinases and anti-breast cancer drugs.

## 3. Materials and Methods

### 3.1. Chemistry

Melting points were determined in an “Electro Thermal” Digital melting point apparatus (Shimadzu, Tokyo, Japan), (model: IA9100). Elemental analysis was performed within the acceptable limits of the calculated values (Microanalytical Unit, NRC). Infrared spectra (KBr) were recorded on a Nexus 670 FTIR Nicolet, Fourier Transform infrared spectrometer (Perkin Elmer, Hopkinton, MA, USA). ^1^H-NMR spectra were run in CDCl_3_ on Jeol 500 MHz instruments (Jeol, Tokyo, Japan). Chemical shifts d are given in ppm. Mass spectra were run on a MAT Finnigan SSQ 7000 spectrometer (Shimadzu, Kyoto, Japan; Model: QP2010 ultra), using the electron impact technique (EI). Analytical thin layer chromatography (TLC) was performed on silica gel aluminum sheets, 60 F254 (E. Merck, Darmstadt, Germany).

#### 3.1.1. Synthesis of Aryl-estra-tetraien-[17,16-*d*]pyrimidinol Derivatives **3a**,**b** and **4a**,**b**

A mixture of **2a**,**b** (10 mmol) [33,34,35] and urea or thiourea (12 mmol) in sodium ethoxide solution [absolute ethanol (25 mL) in the presence of sodium metal (920 mg, 40 mmol)] was refluxed for 5 hrs. The reaction mixture was evaporated under reduced pressure to dryness, and the obtained residue was solidified with water. The formed solid was filtered off, dried and crystallized from methanol/ethyl acetate to give the corresponding **3a**,**b** and **4a**,**b**, respectively.

*2′-Oxo-6′-(2-chlorophenyl)-estra-1(10),2,4,16(17)-tetraien-[17,16-d]pyrimidino-3-ol* (**3a**). Yield 76%, mp 266–268 °C, [*α*]${}_{D}^{25}$ = +104 (*c* 1, MeOH). IR (KBr, cm^−1^): ν = 3450 (OH), 3350 (NH), 3071 and 3055 (CH, aromatic), 2955 (CH, aliphatic), 1720 (C=O), 1644 (C=C). ^1^H-NMR: (500 MHz, ppm, CDCl_3_): δ = 0.61–0.63 (1H, m, CH), 0.94 (3H, s, CH_3_), 1.03–1.62 (6H, m, 6CH), 1.70–1.73 (1H, m, CH), 2.04–2.44 (3H, m, 3CH), 2.52–2.55 (1H, m, CH), 2.62 (1H, s, CH pyrimidine), 2.68–2.70 (1H, m, CH), 4.76 (1H, s, OH, exchangeable with D_2_O), 6.74–7.14 (3H, m, Ar-H), 7.32–7.47 (4H, m, Ar-H), 8.54 (2H, brs, 2NH, exchangeable with D_2_O). ^13^C-NMR: (125 MHz, ppm, CDCl_3_): δ = 13.9, 22.3, 26.6, 26.7, 29.3, 35.78, 39.8, 44.8, 46.3, 47.8, 51.5, 113.5, 115.8, 126.4, 127.9, 128.6, 129.7, 129.8, 133.2, 134.6, 137.3, 138.8, 154.6, 157.7, 158.8, 159.3 (26 C). MS (EI, 70 eV): *m*/*z* (%) = 435 (100%) [M^+^]. Analysis for C_26_H_27_ClN_2_O_2_ (434.95): Calcd C, 71.80; H, 6.26; Cl, 8.17; N, 6.44. Found C, 71.70; H, 6.20; Cl, 8.14; N, 6.38.

*Oxo-6′-(4-chlorophenyl)-estra-1(10),2,4,16(17)-tetraien-[17,16-d]pyrimidino-3-ols* (**3b**). Yield 68%, mp 308–310 °C, [*α*]${}_{D}^{25}$ = +169 (*c* 1, MeOH). IR (KBr, cm^−1^): ν = 3450 (OH), 3358 (NH), 3079 and 3058 (CH, aromatic), 2956 (CH, aliphatic), 1720 (C=O), 1645 (C=C). ^1^H-NMR: (500 MHz, ppm, CDCl_3_): δ = 0.62–0.64 (1H, m, CH), 0.92 (3H, s, CH_3_), 1.01–1.62 (6H, m, 6CH), 1.69–1.72 (1H, m, CH), 2.02–2.44 (3H, m, 3CH), 2.53–2.56 (1H, m, CH), 2.62 (1H, s, CH pyrimidine), 2.70–2.73 (1H, m, CH), 4.66 (1H, s, OH, exchangeable with D_2_O), 6.78–7.17 (3H, m, Ar-H), 7.35–7.55 (4H, m, Ar-H), 8.54 (2H, brs, 2NH, exchangeable with D_2_O). ^13^C-NMR: (125 MHz, ppm, CDCl_3_): δ = 13.4, 22.9, 26.5, 26.8, 29.5, 35.6, 39.3, 44.6, 46.3, 47.1, 51.8, 113.5, 115.5, 126.6, 128.0, 129.4, 133.6, 135.5, 138.6, 138.8, 154.3, 157.9, 158.4, 159.3 (26 C). MS (EI, 70 eV): *m*/*z* (%) = 435 (100%) [M^+^]. Analysis for C_26_H_27_ClN_2_O_2_ (434.95): Calcd C, 71.80; H, 6.26; Cl, 8.17; N, 6.44. Found C, 71.71; H, 6.21; Cl, 8.15; N, 6.37.

*2′-Thione-6′-(2-chlorophenyl)-estra-1(10),2,4,16(17)-tetraien-[17,16-d]pyrimidine-3-ol* (**4a**). Yield 80%, mp 290–292 °C, [*α*]${}_{D}^{25}$ = +135 (*c* 1, MeOH). IR (KBr, cm^−1^): ν = 3458 (OH), 3350 (NH), 3065 and 3047 (CH, aromatic), 2948 (CH, aliphatic), 1637 (C=C), 1614 (C=N), 1050 (C=S). ^1^H-NMR: (500 MHz, ppm, CDCl_3_): δ = 0.60–0.64 (1H, m, CH), 0.91 (3H, s, CH_3_), 1.00–1.61 (6H, m, 6CH), 1.70–1.72 (1H, m, CH), 2.02–2.41 (3H, m, 3CH), 2.49–2.54 (1H, m, CH), 2.62 (1H, s, CH pyrimidine), 2.65–2.67 (1H, m, CH), 4.88 (1H, s, OH, exchangeable with D_2_O), 6.75–7.15 (3H, m, Ar-H), 7.30–7.45 (4H, m, Ar-H), 8.57 (2H, brs, 2NH, exchangeable with D_2_O). ^13^C-NMR: (125 MHz, ppm, CDCl_3_): δ = 13.5, 22.5, 26.6, 26.8, 29.8, 35.7, 39.2, 44.4, 46.1, 47.7, 51.9, 113.4, 115.8, 126.4, 127.9, 128.2, 129.5, 129.6, 133.5, 134.2, 137.7, 138.4, 154.6, 157.6, 158.5, 174.3 (26 C). MS (EI, 70 eV): *m*/*z* (%) = 451 (100%) [M^+^]. Analysis for C_26_H_27_ClN_2_OS (451.02): Calcd C, 69.24; H, 6.03; Cl, 7.86; N, 6.21; S, 7.11. Found C, 69.15; H, 6.00; Cl, 7.80; N, 6.18; S, 7.05.

*2’-Thione-6’-(4-chlorophenyl)-estra-1(10),2,4,16(17)-tetraien-[17,16-d]-pyrimidine-3-ol* (**4b**). Yield 81%, mp 333–335 °C, [*α*]${}_{D}^{25}$ = +144 (*c* 1, MeOH). IR (KBr, cm^−1^): ν = 3448 (OH), 3340 (NH), 3068 and 3047 (CH, aromatic), 2948 (CH, aliphatic), 2558 (SH), 1638 (C=C), 1618 (C=N), 1050 (C=S). ^1^H-NMR: (500 MHz, ppm, CDCl_3_): δ = 0.61–0.64 (1H, m, CH), 0.91 (3H, s, CH_3_), 1.01–1.60 (6H, m, 6CH), 1.70–1.75 (1H, m, CH), 2.01–2.41 (3H, m, 3CH), 2.54–2.56 (1H, m, CH), 2.64 (1H, s, CH pyrimidine), 2.68–2.72 (1H, m, CH), 4.65 (1H, s, OH, exchangeable with D_2_O), 6.74–7.16 (3H, m, Ar-H), 7.36–7.51 (4H, m, Ar-H), 8.54 (2H, s, 2NH, exchangeable with D_2_O). ^13^C-NMR: (125 MHz, ppm, CDCl_3_): δ = 13.3, 22.9, 26.8, 26.9, 29.9, 35.4, 39.2, 44.6, 46.3, 47.1, 51.9, 113.7, 115.6, 126.5, 128.3, 129.4, 133.7, 135.6, 138.7, 138.9, 154.3, 157.9, 158.3, 174.3 (26 C). MS (EI, 70 eV): *m*/*z* (%) = 451 (90%) [M^+^]. Analysis for C_26_H_27_ClN_2_OS (451.02): Calcd C, 69.24; H, 6.03; Cl, 7.86; N, 6.21; S, 7.11. Found C, 69.14; H, 6.01; Cl, 7.79; N, 6.17; S, 7.06.

#### 3.1.2. Synthesis of Imino-6′-aryl-estra-tetraien[17,16-*d*]pyrimidinool Derivatives **5a**,**b** and **6a**,**b**

A mixture of **2a**,**b** (10 mmol) and guanidine hydrochloride or 2-cyanoguanidine (12 mmol) in absolute ethanol (25 mL) in the presence of sodium metal (920 mg, 40 mmol, in 10 mL absolute ethanol) was refluxed for 4–6 h. The reaction mixture was evaporated under reduced pressure, and the obtained residue was washed with water. The obtained solid was filtered off, washed with water, dried, and crystallized from methanol/ethyl acetate to give the corresponding derivatives **5a**,**b** and **6a**,**b**, respectively.

*2′-Imino-6′-(2-chlorophenyl)-estra-1(10),2,4,16(17)-tetraien-[17,16-d]-pyrimidino-3-ol* (**5a**). Yield 80%, mp 286–288 °C, [*α*]${}_{D}^{25}$ = +135 (*c* 1, MeOH). IR (KBr, cm^−1^): ν = 3458 (OH), 3350–3390 (NH), 3063 and 3047 (CH, aromatic), 2950 (CH, aliphatic), 1638 (C=C), 1614 (C=N). ^1^H-NMR: (500 MHz, ppm, CDCl_3_): δ = 0.61–0.63 (1H, m, CH), 0.90 (3H, s, CH_3_), 1.01–1.59 (6H, m, 6CH), 1.67–1.69 (1H, m, CH), 2.01–2.42 (3H, m, 3CH), 2.50–2.53 (1H, m, CH), 2.62 (1H, s, CH pyrimidine), 2.63–2.66 (1H, m, CH), 4.91 (1H, s, OH, exchangeable with D_2_O), 6.77–7.11 (3H, m, Ar-H), 7.34–7.49 (4H, m, Ar-H), 8.51 (2H, bs, 2NH, exchangeable with D_2_O), 8.82 (1H, bs, NH, exchangeable with D_2_O). ^13^C-NMR: (125 MHz, ppm, CDCl_3_): δ = 13.5, 22.3, 26.2, 26.7, 29.6, 35.9, 44.2, 39.0, 47.1, 47.9, 51.2, 113.3, 115.6, 126.5, 127.8, 128.3, 129.4, 129.5, 133.8, 134.3, 137.8, 138.3, 154.4, 157.8, 158.1, 163.3 (26 C). MS (EI, 70 eV): *m*/*z* (%) = 434 (100%) [M^+^]. Analysis for C_26_H_28_ClN_3_O (433.97): Calcd C, 71.96; H, 6.50; Cl, 8.17; N, 9.68. Found C, 71.90; H, 6.45; Cl, 8.11; N, 9.62.

*2′-Imino-6′-(4-chlorophenyl)-estra-1(10),2,4,16(17)-tetraien-[17,16-d]pyrimidino-3-ol* (**5b**). Yield 81%, mp 326–328 °C, [*α*]${}_{D}^{25}$ = +144 (*c* 1, MeOH). IR (KBr, cm^−1^): ν = 3458 (OH), 3350–3390 (NH), 3060 and 3040 (CH, aromatic), 2951 (CH, aliphatic), 1636 (C=C), 1617 (C=N). ^1^H-NMR: (500 MHz, ppm, CDCl_3_): δ = 0.62–0.64 (1H, m, CH), 0.93 (3H, s, CH_3_), 1.02–1.61 (6H, m, 6CH), 1.66–1.69 (1H, m, CH), 2.01–2.41 (3H, m, 3CH), 2.51–2.54 (1H, m, CH), 2.64 (1H, s, CH pyrimidine), 2.71–2.75 (1H, m, CH), 4.88 (1H, s, OH, exchangeable with D_2_O), 6.76–7.16 (3H, m, Ar-H), 7.36–7.51 (4H, m, Ar-H), 8.49 (2H, brs, 2NH, exchangeable with D_2_O), 8.80 (1H, brs, NH, exchangeable with D_2_O). ^13^C-NMR: (125 MHz, ppm, CDCl_3_): δ = 13.3, 22.4, 26.4, 26.5, 29.8, 35.6, 39.3, 44.8, 47.3, 47.5, 51.7, 113.5, 115.6, 126.3, 128.3, 129.4, 133.5, 135.6, 138.6, 138.9, 154.6, 157.5, 158.3, 163.6 (26 C). MS (EI, 70 eV): *m*/*z* (%) = 434 (100%) [M^+^]. Analysis for C_26_H_28_ClN_3_O (433.97): Calcd C, 71.96; H, 6.50; Cl, 8.17; N, 9.68. Found C, 71.91; H, 6.44; Cl, 8.10; N, 9.63.

*2′-Cyanoimino-6′-(2-chlorophenyl)-estra-1(10),2,4,16(17)-tetraien-[17,16-d]pyrimidino-3-ol* (**6a**). Yield 67%, mp 223 °C, [*α*]${}_{D}^{25}$ = +91 (*c* 1, MeOH). IR (KBr, cm^−1^): ν = 3461 (OH), 3358–3391 (NH), 3064 and 3045 (CH, aromatic), 2956 (CH, aliphatic), 2225(CN), 1637 (C=C), 1618 (C=N). ^1^H-NMR: (500 MHz, ppm, CDCl_3_): δ = 0.62–0.64 (1H, m, CH), 0.91 (3H, s, CH_3_), 1.01–1.56 (6H, m, 6CH), 1.65–1.68 (1H, m, CH), 2.00–2.40 (3H, m, 3CH), 2.48–2.52 (1H, m, CH), 2.61 (1H, s, CH pyrimidine), 2.64–2.68 (1H, m, CH), 4.91 (1H, s, OH, exchangeable with D_2_O), 6.79–7.12 (3H, m, Ar-H), 7.32–7.47 (4H, m, Ar-H), 9.87 (2H, brs, 2NH, exchangeable with D_2_O). ^13^C-NMR: (125 MHz, ppm, CDCl_3_): δ = 13.3, 22.3, 26.5, 26.7, 29.6, 35.4, 39.3, 44.2, 47.6, 48.3, 51.7, 113.3, 115.6, 126.5, 128.1, 128.5, 129.1, 129.2, 133.5, 134.4, 137.8, 138.3, 154.4, 157.6, 158.7, 163.6, 172.1 (27 C). MS (EI, 70 eV): *m*/*z* (%) = 459 (85%) [M^+^]. Analysis for C_27_H_27_ClN_4_O (458.98): Calcd C, 70.65; H, 5.93; Cl, 7.72; N, 12.21. Found C, 70.60; H, 5.90; Cl, 7.68; N, 12.17.

*2′-Cyanoimino-6′-(4-chlorophenyl)-estra-1(10),2,4,16(17)-tetraien-[17,16-d]-pyrimidino-3-ol* (**6b**). Yield 77%, mp 282–284 °C, [*α*]${}_{D}^{25}$ = +121 (*c* 1, MeOH). IR (KBr, cm^−1^): ν = 3461 (OH), 3355–3394 (NH), 3065 and 3046 (CH, aromatic), 2957 (CH, aliphatic), 2228(CN), 1638 (C=C), 1619 (C=N). ^1^H-NMR: (500 MHz, ppm, CDCl_3_): δ = 0.60–0.65 (1H, m, CH), 0.92 (3H, s, CH_3_), 1.01–1.60 (6H, m, 6CH), 1.70Synthesis of Substituted phenyl-estratrien1.75 (1H, m, CH), 1.98–2.45 (3H, m, 3CH), 2.52–2.56 (1H, m, CH), 2.60 (1H, s, CH pyrimidine), 2.70–2.75 (1H, m, CH), 4.79 (1H, s, OH, exchangeable with D_2_O), 5.76–7.16 (3H, m, Ar-H), 7.35–7.46 (4H, m, Ar-H) 9.87 (2H, brs, 2NH, exchangeable with D_2_O). ^13^C-NMR: (125 MHz, ppm, CDCl_3_): δ = 13.4, 22.2, 26.4, 26.5, 29.6, 35.5, 39.3, 44.5, 47.4, 48.7, 51.6, 113.6, 115.7, 126.4, 129.1, 128.1, 133.5, 135.2, 138.6, 138.8, 154.6, 157.5, 158.1, 163.6, 172.2 (27 C). MS (EI, 70 eV): *m*/*z* (%) = 459 (90%) [M^+^]. Analysis for C_27_H_27_ClN_4_O (458.98): Calcd C, 70.65; H, 5.93; Cl, 7.72; N, 12.21. Found C, 70.58; H, 5.91; Cl, 7.68; N, 12.16.

#### 3.1.3. Synthesis of Substituted Phenyl-estratrien[17,16-*c*]pyrazoline Derivatives **7a**,**b** and **8a**,**b**

A solution of derivatives **2a**,**b** (10 mmol), semicarbazide or thiosemicarbazide (12 mmol) in dioxane (25 mL) was refluxed for 5–7 h. The reaction mixture was evaporated to dryness under vacuum, then washed with water. The obtained precipitate was filtered off, washed with water, dried and crystallized from methanol/methyl acetate to give the corresponding compounds **7a**,**b** and **8a**,**b**, respectively. 

*1-Carbamoyl-1′H-(2’H)-5′-(2-chlorophenyl)-estra-1(10),2,4-trien-[17,16-c]pyrazoline-3-ol* (**7a**). Yield 58%, mp 298–300 °C, [*α*]${}_{D}^{25}$ = +155 (*c* 1, MeOH). IR (KBr, cm^−1^): ν = 3351(OH), 3285 (NH_2_), 3060 and 3050 (CH, aromatic), 2941 (CH, aliphatic), 1688 (C=O), 1642 (C=C), 1622 (C=N). ^1^H-NMR: (500 MHz, ppm, CDCl_3_): δ = 0.62–0.68 (1H, m, CH), 0.92 (3H, s, CH_3_), 1.10–1.60 (6H, m, 6CH), 1.70–1.76 (1H, m, CH), 2.05–2.24 (3H, m, 3CH), 2.42–2.47 (1H, m, CH), 2.54–2.58 (1H, m, CH), 2.66–2.70 (1H, m, CH), 3.87 (1H, s, CH pyrazoline), 4.86 (1H, s, OH, exchangeable with D_2_O), 6.42–7.10 (3H, m, Ar-H), 7.27–7.40 (4H, m, Ar-H), 9.65 (2H, bs, NH_2_, exchangeable with D_2_O). ^13^C-NMR: (125 MHz, ppm, CDCl_3_): δ = 13.4, 22.5, 26.4, 26.9, 28.7, 30.5, 36.6, 39.8, 49.1, 44.7, 51.2, 64.5, 113.2, 115.6, 127.6, 127.9, 128.6, 129.7, 129.8, 133.4, 134.6, 137.6, 138.5, 154.6, 163.6, 164.6 (26 C). MS (EI, 70 eV): *m*/*z* (%) = 450 (100%) [M^+^]. Analysis for C_26_H_28_ClN_3_O_2_ (449.97): Calcd C, 69.40; H, 6.27; Cl, 7.88; N, 9.34. Found C, 69.30; H, 6.22; Cl, 7.83; N, 9.30.

*1-Carbamoyl-1’H-(2’H)-5’-(4-chlorophenyl)-estra-1(10),2,4-trien-[17,16-c]pyrazoline-3-ol* (**7b**). Yield 53%, mp 248–250 °C, [*α*]${}_{D}^{25}$ = +126 (*c* 1, MeOH). IR (KBr, cm^−1^): ν = 3354(OH), 3284 (NH_2_), 3064 and 3055 (CH, aromatic), 2946 (CH, aliphatic), 1687 (C=O), 1648 (C=C), 1629 (C=N). ^1^H-NMR: (500 MHz, ppm, CDCl_3_): δ = 0.63–0.65 (1H, m, CH), 0.94 (3H, s, CH_3_), 1.02–1.64 (6H, m, 6CH), 1.73–1.75 (1H, m, CH), 2.04–2.31 (3H, m, 3 CH), 2.42–2.46 (1H, m, CH), 2.54–2.60 (1H, m, CH), 2.70–2.75 (1H, m, CH), 3.90 (1H, s, CH pyrazoline), 4.77 (1H, s, OH, exchangeable with D_2_O), 6.74–7.11 (3H, m, Ar-H), 7.31–7.51 (4H, m, Ar-H), 10.08 (2H, bs, NH_2_, exchangeable with D_2_O). ^13^C-NMR: (125 MHz, ppm, CDCl_3_): δ = 13.3, 22.5, 26.4, 26.6, 28.8, 30.6, 36.4, 39.5, 44.5, 49.6, 51.5, 64.6, 113.3, 115.8, 127.5, 128.4, 129.5, 133.5, 135.4, 138.5, 138.7, 154.7, 163.4, 164.9 (26 C). MS (EI, 70 eV): *m*/*z* (%) = 450 (80%) [M^+^]. Analysis for C_26_H_28_ClN_3_O_2_ (449.97): Calcd C, 69.40; H, 6.27; Cl, 7.88; N, 9.34. Found C, 69.34; H, 6.22; Cl, 7.82; N, 9.31.

*1-Thiocarbamoyl-1′H-(2′H)-5′-(2-chlorophenyl)-estra-1(10),2,4-trien-[17,16-c]pyrazoline-3-ol* (**8a**). Yield 58%, mp 294–296 °C, [*α*]${}_{D}^{25}$ = +155 (*c* 1, MeOH). IR (KBr, cm^−1^): ν = 3351(OH), 3285 (NH_2_), 3060 and 3050 (CH, aromatic), 2941 (CH, aliphatic), 1642 (C=C), 1622 (C=N), 1550 (C=S). ^1^H-NMR: (500 MHz, ppm, CDCl_3_): δ = 0.64–0.66 (1H, m, CH), 0.93 (3H, s, CH_3_), 1.04–1.62 (6H, m, 6CH), 1.74–1.76 (1H, m, CH), 2.07–2.25 (3H, m, 3CH), 2.44–2.46 (1H, m, CH), 2.58–2.60 (1H, m, CH), 2.68–2.72 (1H, m, CH), 3.88 (1H, s, CH pyrazoline), 4.87 (1H, s, OH, exchangeable with D_2_O), 6.77–7.10 (3H, m, Ar-H), 7.28–7.41 (4H, m, Ar-H), 9.85 (2H, bs, NH_2_, exchangeable with D_2_O). ^13^C-NMR: (125 MHz, ppm, CDCl_3_): δ = 13.7, 22.5, 26.6, 26.9, 28.7, 30.6, 36.9, 39.8, 44.7, 49.1, 51.2, 64.8, 113.1, 115.7, 127.3, 127.8, 128.5, 129.4, 129.5, 133.7, 134.5, 137.9, 138.8, 154.6, 163.6, 194.6 (26 C). MS (EI, 70 eV): *m*/*z* (%) = 466 (100%) [M^+^]. Analysis for C_26_H_28_ClN_3_OS (466.04): Calcd C, 67.01; H, 6.06; Cl, 7.61; N, 9.02; S, 6.88. Found C, 66.98; H, 6.00; Cl, 7.57; N, 9.00; S, 6.84.

*1-Thiocarbamoyl-1′H-(2′H)-5′-(4-chlorophenyl)-estra-1(10),2,4-trien-[17,16-c]pyrazoline-3-ol* (**8b**). Yield 53%, mp 252–256 °C, [*α*]${}_{D}^{25}$ = +126 (*c* 1, MeOH). IR (KBr, cm^−1^): ν = 3353 (OH), 3285 (NH_2_), 3064 and 3055 (CH, aromatic), 2946 (CH, aliphatic), 1647 (C=C), 1628 (C=N), 1559 (C=S). ^1^H-NMR: (500 MHz, ppm, CDCl_3_): δ = 0.65–0.67 (1H, m, CH), 0.94 (3H, s, CH_3_), 1.06–1.68 (6H, m, 6CH), 1.76–1.78 (1H, m, CH), 2.09–2.28 (3H, m, 3CH), 2.47–2.49 (1H, m, CH), 2.59–2.61 (1H, m, CH), 2.70–2.72 (1H, m, CH), 3.88 (1H, s, CH pyrazoline), 4.90 (1H, s, OH, exchangeable with D_2_O), 6.80–7.13 (3H, m, Ar-H), 7.33–7.49 (4H, m, Ar-H), 10.11 (2H, bs, NH_2_, exchangeable with D_2_O). ^13^C-NMR: (125 MHz, ppm, CDCl_3_): δ = 13.3, 22.6, 26.4, 26.8, 28.9, 30.9, 36.5, 39.5, 44.6, 49.7, 51.5, 64.6, 113.9, 115.5, 127.7, 128.5, 129.6, 133.7, 135.5, 138. 7, 138.8, 154.9, 163.5, 194.9 (26 C). MS (EI, 70 eV): *m*/*z* (%) = 466 (100, M^+^]. Analysis for C_26_H_28_ClN_3_OS (466.04): Calcd C, 67.01; H, 6.06; Cl, 7.61; N, 9.02; S, 6.88. Found C, 66.94; H, 6.00; Cl, 7.56; N, 9.00; S, 6.83.

### 3.2. Biological Evaluation

#### 3.2.1. In Vitro Cytotoxic Activity against MCF-7 Cancer Cells

MCF-7 (human breast cancer) cells, obtained from Sigma-Aldrich Chemie GmbH, Taufkirchen, Germany, were propagated in RPMI-1640 supplemented with 10% heat inactivated FBS, 2 mM l-glutamine, and 1% standard antibiotic solution. Cells were incubated in a 5% CO_2_ humidified incubator at 37 °C and passaged bi-weekly. The in vitro anti-proliferative activity of the newly synthesized derivatives was assayed using standard MTT technique [39,41,42]. Results were expressed as IC_50_. Experiments were repeated at least in triplicate, to obtain good reproducibility between replicate wells with standard errors below 10%. Furthermore, the cytotoxic effects of the prepared derivatives were evaluated against normal nonmalignant cells, non-tumorigenic MCF-10A, in order to find out if the synthesized derivatives have toxicity against normal cells. Additionally, results were compared with reference compounds (cisplatin and milaplatin) as positive controls.

#### 3.2.2. In Vivo Human Breast Cancer Xenograft Models and Animal Treatment

The breast cancer xenograft model protocol [24] was approved by the Institutional Animal Use and Care Committee of the University of Alabama at Birmingham (50-01-05-08B). Female athymic pathogen-free nude mice (nu/nu, 4–6 weeks) were used. Firstly, MCF-7 xenografts were initiated by implanting pellets slowly releasing estrogen for two months (1.7mg 17β-estradiol/pellet) subcutaneously in the female nude mice. After 24 h, confluent MCF-7 cells were harvested, washed two times with serum-free medium, re-suspended and injected subcutaneously (s.c.) (5 × 10^6^ cells, total volume 0.2 mL) into the left inguinal area of the mice. Caliper measurement was used to measure tumor growth in two perpendicular diameters of the implant after 48 h, and its volume was determined [43]. Mice grafted with MCF-7 were divided into different groups (7–10 mice/group). Untreated mice received the solvent only. Treated groups received different prepared derivatives as previously described [24].

#### 3.2.3. In Vitro p53 Ubiquitination Assay

Different prepared derivatives were evaluated for their inhibitory potential against p53 ubiquitination. The method is based on incubating different compounds with GST-tagged HDM2, immobilized on glutathione-Sepharose, p53, ubiquitin, E1 and E2 (UbcH5B) ligases. The buffer contained ATP as detailed by Gomha and Abdelaziz [44]. The reaction products were then resolved by SDSPAGE and p53 ubiquitination and were quantified by Western blotting using an anti-p53 antibody [45].

Briefly, rGSH-S-transferase-tagged MDM2 (GST-MDM2) was expressed in *Escherichia coli* BL21 cells using 1 mM isopropyl-thio-b-d-galactoside for 3 h. The enzyme was then purified on glutathione-sepharose beads (Amersham). Beads were washed with Tris (pH 7.5). The reaction contained fluorescent ubiquitin (5 µg; Invitrogen), 50 ng mammalian E1 (Enzo), 200 ng human rUbcH5B E2 (Enzo), 200 ng His-p53 (Enzo) and buffer [50 mM Tris pH 8,2 mM dithiothreitol, 5 mM MgCl_2_, 2 mM adenosine triphosphate (ATP)]. The MPD-compound or control mixture was dose-titrated onto GS4b-MDM2 beads. Incubation at 37 °C was followed by shaking (1200 rpm, 1 h). The reaction was terminated with 3× sodium dodecyl sulfate sample buffer. Finally, unbound fluorescent ubiquitin was drained and the total fluorescent ubiquitin signal was measured using a plate reader (Safire).

#### 3.2.4. In Vivo Ubiquitination of p53

In vivo ubiquitination of p53 was assayed according to [38] Cells were grown for 24 h (50% confluency) before transfection of 1 µg p53, 4 µg MDM2 and 1 µg HIS-ubiquitin with Gene Juice reagent. Grown for another 20 h, cells were exposed to prepared derivatives or controls for 6 h and MG132 for 4 h, then washed and collected in PBS, where 5% of the solution was kept as an input. The remaining pelleted cells (95% after centrifugation) were lysed in 700 µL of ubiquitin buffer A (6 M guanidinium HCl, 300 mM NaCl, 50 mM phosphate pH 8.0, 100 µg/mL N-ethylmaleimide), and sonicated for 5 min at 20% amplitude. Lysates were incubated overnight with Invitrogen Dynabeads His-Tag matrix and washed once with ubiquitin buffers A, B, C and phosphate buffered saline (ubiquitin buffer B: Mix ubiquitin buffer A and ubiquitin buffer C 1:1; ubiquitin buffer C: 300 mM NaCl, 50 mM phosphate pH 8.0, 100 µg/mL N-ethylmaleimide). The washed lysates were resolved by 8% sodium dodecyl sulfate–polyacrylamide gel electrophoresis, followed by immunoblotting with a p53 (DO-1) antibody.

#### 3.2.5. Non-Fluorescent In Vitro Ubiquitination

The procedure was performed similar to the aforementioned protocol except that 5 µg unlabeled ubiquitin (Enzo) was used. Following incubation, resolving of reaction products was performed using sodium dodecyl sulfate PGE. Analysis was done by western blotting using anti-p53 DO-1. For the MDM2 RING auto-ubiquitination assay, GS4b-MDM2 RING beads were prepared as above and were used in place of the full-length MDM2.

#### 3.2.6. Determination of EGFR and VEGFR-2 Kinase Activities

The EGFR and VEGFR-2 kinase activities were assayed as previously described [39]. The assay was performed in 96-well plates pre-coated with 20 μg mL^−1^ poly (Glu, Tyr)4:1 (Sigma-Aldrich Chemie GmbH, Taufkirchen, Germany) as a substrate. In each well, 85 μL of an 8 μM ATP solution and 10 μL of the compound were added at varying concentrations. Experiments were repeated three times. Reactions started with 5 μL of different kinases and incubation for 1 h at 37 °C. Plates were 3X-washed with PBS/0.1% Tween 20. Accordingly, 100 μL of anti-phosphotyrosine (PY99; 1:500 dilutions) antibody was added, and plates were incubated for another hour at room temperature. Plates were then 3X-washed, and addition of goat anti-mouse IgG horseradish peroxidase (100 μL; 1:2000 dilution) diluted in T-PBS containing 5 mg mL^−1^ BSA followed. Plates were further incubated at room temperature for another hour, and then 3X-washed. Finally, 100 μL (developing solution, 0.03% H_2_O_2_, 2 mg mL^−1^ o-phenylenediamine/citrate buffer, 0.1 M, pH 5.5) was added and incubation continued until color development. The reaction was stopped by 100 μL of 2M H_2_SO_4_, and the absorption was measured at 492 nm using a multiwell spectrophotometer (VERSAmax™). The inhibition rate (%) was calculated using the equation:

Inhibition rate (%) = [1 − (A_492_/A_492Control_)] × 100% (1)

#### 3.2.7. Statistical Analysis

Results are expressed as the mean ± standard error of mean (SEM), *n* = 6 in each group. Statistical analysis was performed by one way analysis of variance (ANOVA) followed by Dunnet’s test using Graphpad Instat software, *p* < 0.05.

### 3.3. Molecular Modeling Studies

Molecular docking simulation was carried out using Molecular Operating Environment (MOE, 10.2008) software according to the reported method [39]. The three-dimensional X-ray structures of EGFR (PDB code: 1M17) [40] and VEGFR-2 (PDB code: 4ASD) [39] were obtained from the Protein Data Bank through the Internet.

## 4. Conclusions

In this study, a new series of estrone derivatives **3**–**8** was synthesized by heterocyclization at ring D of the steroidal moiety. All new compounds were evaluated for their in vitro and in vivo inhibitory activities against breast cancer, EGFR and VEGFR-2 kinases and p53 ubiquitination. Depending on their structural substitution, it was clear that all derivatives revealed promising and variable inhibitory activities following a similar descending order **5a**, **5b**, **6a**, **6b**, **4a**, **4b**, **3a**, **3b**, **8a**, **8b**, **7a** and **7b** in comparison to the known standard drugs (diphenyl imidazole and delphinidin). Such a finding was justified by molecular docking of the most potent compound **5a**, which represented the importance of molecular hybridization of estrone with pyrimidine moiety at ring D, and this affords a new promising chemotherapeutic candidate that could be optimized for further development of potent anti-breast cancer agents.

## Figures and Tables

**Table 1:** In vitro cytotoxic activities of the tested compounds **3**–**8** against MCF-7 cell lines using MTT assay.

Compound No.	IC_50_ (Mean ± SEM) (µM)	
	MCF-7	MCF-10A
**3a**	35.66 ± 0.40	401.23 ± 11.56
**3b**	38.57 ± 0.87	422.33 ± 13.76
**4a**	28.64 ± 0.28	301.54 ± 8.86
**4b**	31.75 ± 0.39	310.56 ± 9.59
**5a**	18.38 ± 0.13	211.23 ± 7.53
**5b**	22.48 ± 0.24	234.56 ± 7.76
**6a**	24.57 ± 0.16	243.54 ± 7.78
**6b**	26.46 ± 0.17	276.87 ± 8.23
**7a**	41.12 ± 0.84	465.77 ± 17.34
**7b**	46.17 ± 0.93	512.34 ± 16.65
**8a**	39.46 ± 0.75	434.65 ± 14.57
**8b**	40.35 ± 0.95	445.67 ± 15.43
**Cisplatin**	13.34 ± 0.11	9.55 ± 0.54
**Milaplatin**	18.43 ± 0.13	18.30 ± 0.34

IC_50_: Compound concentration required to inhibit the cell viability by 50%, SEM = standard error of the mean; each value is the mean of three values; *n* = 6 in each group; Statistical analysis by one way analysis of variance (ANOVA) followed by Dunnet’s test using Graphpad Instat software (*p* < 0.05).

**Table 2:** In vitro and in vivo p53 ubiquitination of the newly synthesized compounds **3**–**8**.

Compound No.	IC_50_ (Mean ± SEM) (µM)	
	In Vitro p53 Ubiquitination	In Vivo p53 Ubiquitination
**3a**	47.65 ± 0.60	0.60 ± 0.0086
**3b**	53.56 ± 0.78	0.63 ± 0.0095
**4a**	36.64 ± 0.58	0.45 ± 0.0085
**4b**	45.76 ± 0.49	0.57 ± 0.0097
**5a**	16.45 ± 0.23	0.22 ± 0.0043
**5b**	27.56 ± 0.34	0.31 ± 0.0053
**6a**	28.47 ± 0.25	0.32 ± 0.0063
**6b**	29.55 ± 0.36	0.42 ± 0.0074
**7a**	66.65 ± 0.86	0.86 ± 0.0068
**7b**	77.56 ± 0.97	0.89 ± 0.0099
**8a**	55.45 ± 0.69	0.74 ± 0.0087
**8b**	64.56 ± 0.78	0.82 ± 0.0078
**Diphenyl Imidazole**	260 ± 0.02	1.88 ± 0.0047

IC_50_: Compound concentration required to inhibit p53 ubiquitination by 50%, SEM = standard error of the mean; each value is the mean of three values; *n* = 6 in each group; Statistical analysis by one way analysis of variance (ANOVA) followed by Dunnet’s test using Graphpad Instat software (*p* < 0.05).

**Table 3:** Enzymatic inhibitory evaluation of all synthesized compounds **3**–**8** against VEGFR-2 and EGFR.

Compound No.	IC_50_ (Mean ± SEM) (µM)	
	EGFR	VEGFR-2
**3a**	0.127 ± 0.0037	0.047 ± 0.003
**3b**	0.155 ± 0.0051	0.048 ± 0.0014
**4a**	0.095 ± 0.0028	0.037 ± 0.0026
**4b**	0.098 ± 0.0009	0.043 ± 0.0018
**5a**	0.086 ± 0.0032	0.027 ± 0.0012
**5b**	0.088 ± 0.0013	0.029 ± 0.0003
**6a**	0.090 ± 0.0006	0.031 ± 0.0041
**6b**	0.091 ± 0.0025	0.033 ± 0.0010
**7a**	0.188 ± 0.0016	0.055 ± 0.0013
**7b**	0.227 ± 0.0004	0.057 ± 0.0005
**8a**	0.166 ± 0.0028	0.051 ± 0.0017
**8b**	0.177 ± 0.0041	0.053 ± 0.0023
**Delphinidin**	6.27 ± 0.00076	5.09 ± 0.0012

IC_50_: Compound concentration required to inhibit the enzyme activity by 50%, SEM = standard error of the mean; each value is the mean of three values; *n* = 6 in each group; Statistical analysis by one way analysis of variance (ANOVA) followed by Dunnet’s test using Graphpad Instat software (*p* < 0.05).
